# Evaluating Health in All Policies: Comment on "Developing a Framework for a Program Theory-Based Approach to Evaluating Policy Processes and Outcomes: Health in All Policies in South Australia"

**DOI:** 10.15171/ijhpm.2018.33

**Published:** 2018-04-08

**Authors:** Sebastián Peña

**Affiliations:** Department of Public Health Solutions, National Institute for Health and Welfare, Helsinki, Finland.

**Keywords:** Health in All Policies, Health Policy, Policy-Making, Evaluation

## Abstract

Health in All Policies (HiAP) has gained attention as a potential tool to address complex health and societal challenges at global, regional, national and subnational levels. In a recent article, Lawless et al propose an evaluation framework developed in the context of the South Australia HiAP initiative. Strategies, mediators, activities and impacts identified in the framework could potentially be useful for evaluating HiAP in other settings. Creating and sustaining political will, managing conflicts of interest and achieving financially, politically and conceptually sustainable HiAP initiatives are challenges that could be further strengthened in the current framework.

## Evaluating Health in All Policies

The need for effective integration of health and health equity in the policies of various sectors is increasingly recognized. Health in All Policies (HiAP) has gained attention as a potential tool to address complex health and societal challenges at global, regional, national and subnational levels. For the World Health Organization (WHO) Global Health Promotion Conference in 2013, WHO Regional Offices collected a wide range of case studies that used a HiAP approach.^1-3^ Finland, the host, produced a book with a compilation of experiences from all continents, using Kingdon’s framework as a starting point to account for the non-linearity of the policy-making process.^4^ Since then, HiAP literature has significantly expanded, including implementation experiences in national and local jurisdictions, as well as theoretical and discussion pieces.^5-10^ WHO produced a training manual to build capacity to design, implement and evaluate HiAP.^11^

HiAP has been defined as an “approach to public policies across sectors that systematically takes into account the health and health systems implications of decisions, seeks synergies, and avoids harmful health impacts, in order to improve population health and health equity.”^4^ As the definition states, HiAP is not an objective by itself, but rather a tool to improve the quality of policy-making from the health and health equity point of view.

The ambitious task of developing a systematic framework for evaluating HiAP by Lawless et al is timely and highly welcomed.^12^ The framework is based on program theory and departs from a linear understanding of policy-making. To account for the messy, “juggling” process of policy-making, the authors draw on Kingdon’s framework of three streams (problems, policies, politics) and windows of opportunity arising after the convergence of these streams. The framework identifies the assumptions, context, strategies, activities – which includes actors, structures and processes involved in HiAP – that result in outputs and outcomes of HiAP in the State of South Australia.

The framework identifies three strategies worth commenting upon. Two strategies are devoted to building relational and governance systems that connect individuals and actors in an institutional context. Policy-making is, after all, made by people and connecting these people to create a culture of collaboration is essential to build a common ground for HiAP. The essential activities for these strategies include establishing structures, such as the dedicated HiAP unit, and processes (eg, health lens analysis) that allow bureaucrats and senior decision-makers to connect. A similar dedicated structure has been used in the California Health in All Policies Task Force.^13^

The authors denote these structures and processes in the way they have been implemented in South Australia. There are, however, several possible arrangements as documented carefully by McQueen and colleagues, with various degrees of success depending on contextual factors.^14^ A broader conceptualization of structures and processes could strengthen the generalizability of Lawless et al framework. More importantly, dedicated structures and processes put HiAP at risk of transforming it in one more silo if not well balanced with a broad institutional vision of the importance of health in policy-making.^15^

A third strategy draws attention to the issue of framing, or how to articulate a common goal that provides policy gains not only for the health sector but to all sectors. A broad framing that includes the objectives of other (non-health) sectors can potentially provide better momentum to reach synergies and to gain greater political will than asking other sectors to contribute to a health issue (as in the classic concept of *intersectoral action for health*). It could also prevent criticisms of “health imperialism.” For example, Ecuador has framed HiAP issues around the concept of wellbeing, as the agglutinating force of HiAP.^1^ In this view, all sectors contribute to the wellbeing of the population, placing the health sector as a contributor together with education, housing, social services, transport, economy, to name a few.

The issues of politics, political will and resources act as mediators in the current framework, which visually undermine their importance. It is unclear how these mediators interact with the strategies and activities and what would be their relative contribution to the success or failure of the strategies. In their evaluation of HiAP at the local level in Finland, Kokkinen et al identified the dismantlement of a formal funding allocation from the State to municipalities, the power relationships between the State and international institutions and an ideology of deregulation to have played a major role as barriers in achieving the goals of the Health 2015 policy programme.^16^ Politics, political will and resources have been highlighted as major determinants of HiAP in other reports and have received more attention in other evaluations, such as the HARMONICS framework.^4,17^

Conflicts of interest, an emerging theme in the policy-making related to noncommunicable diseases (NCDs), does not seem to have played a role in the South Australia experience. Recent research suggests, however, that conflicts of interest are a key factor preventing governments and subnational jurisdictions to promote cost-effective polities to curb NCDs. The experience of Mexico and Chile in the process of introducing and raising taxes on sugar-sweetened beverages remains as a cautionary tale of the power of the food industry to promote commercial interests over public health.^18^ Similar challenges have been extensively documented in the field of tobacco or alcohol policy.

One of the key challenges in all HiAP initiatives is sustainability. This includes financial sustainability to maintain the structures and processes, the political sustainability to keep the political will from the highest authority and the conceptual sustainability to systematically preserve HiAP in the context of competing agendas and frameworks. Changes in government can be particularly devastating when the HiAP initiatives are not administratively or legally grounded and can result in significant turnover of civil servants in the health and other sectors, as well as a change in routines and engagement mechanisms that proved successful in the past. They can, in turn, provide new windows of opportunity for policy change. The framework could be strengthened by taking sustainability more explicitly into account.

All in all, the article by Lawless et al provides an excellent contribution to the evaluation of HiAP initiatives worldwide. Future research could provide more insights into how existing initiative have been able to create and sustain political will, address conflicts of interest and ensure sustainability over time.

## Ethical issues

Not applicable.

## Competing interests

Author declares that he has no competing interests.

## Author’s contribution

SP is the single author of the paper.
